# Relationship Between Melatonin and Cardiovascular Disease

**DOI:** 10.7759/cureus.12935

**Published:** 2021-01-27

**Authors:** Flora Ozkalayci, Umut Kocabas, Betul U Altun, Seithikurippu Pandi-Perumal, Armagan Altun

**Affiliations:** 1 Cardiology, Hisar Intercontinental Hospital, Istanbul, TUR; 2 Cardiology, Baskent University Izmir Hospital, Izmir, TUR; 3 Endocrinology, Academic Hospital, Istanbul, TUR; 4 Sleep Medicine, Somnogen Canada Inc, Toronto, CAN; 5 Cardiology, Baskent University İstanbul Hospital, Istanbul, TUR

**Keywords:** melatonin, coronary artery disease, cardiovascular, risk factors, circadian rhythm

## Abstract

Coronary artery disease (CAD) is one of the leading causes of morbidity and mortality worldwide. The coronary atherosclerotic process involves different pathological mechanisms; inflammation is one of the major triggers for the development of atherosclerotic plaque. Although several studies showed the favorable effects of melatonin on the cardiovascular system (CVS), melatonin seems not to take its rightful place in today’s clinical practice. This review aims to point out the role of melatonin on cardiovascular disease (CVD) and its’ risk factors. All data were obtained via PubMed, Wikipedia, and Google.

## Introduction and background

Melatonin is a neuroendocrine mediator known to have an impact on several biologic systems such as lipid and glucose metabolism, blood pressure, the sleep-wake system. Disorders of these systems are also related to coronary artery disease (CAD). CAD is one of the leading causes of morbidity and mortality worldwide. While many risk factors have been identified for CAD and its’ prognosis, melatonin seems not to take its right full place in today’s clinical practice. Several studies showed the favourable effects of melatonin on the cardiovascular system (CVS). This review aims to point out the role of melatonin on cardiovascular disease (CVD).

## Review

Melatonin’s structure and function

**Figure 1:**
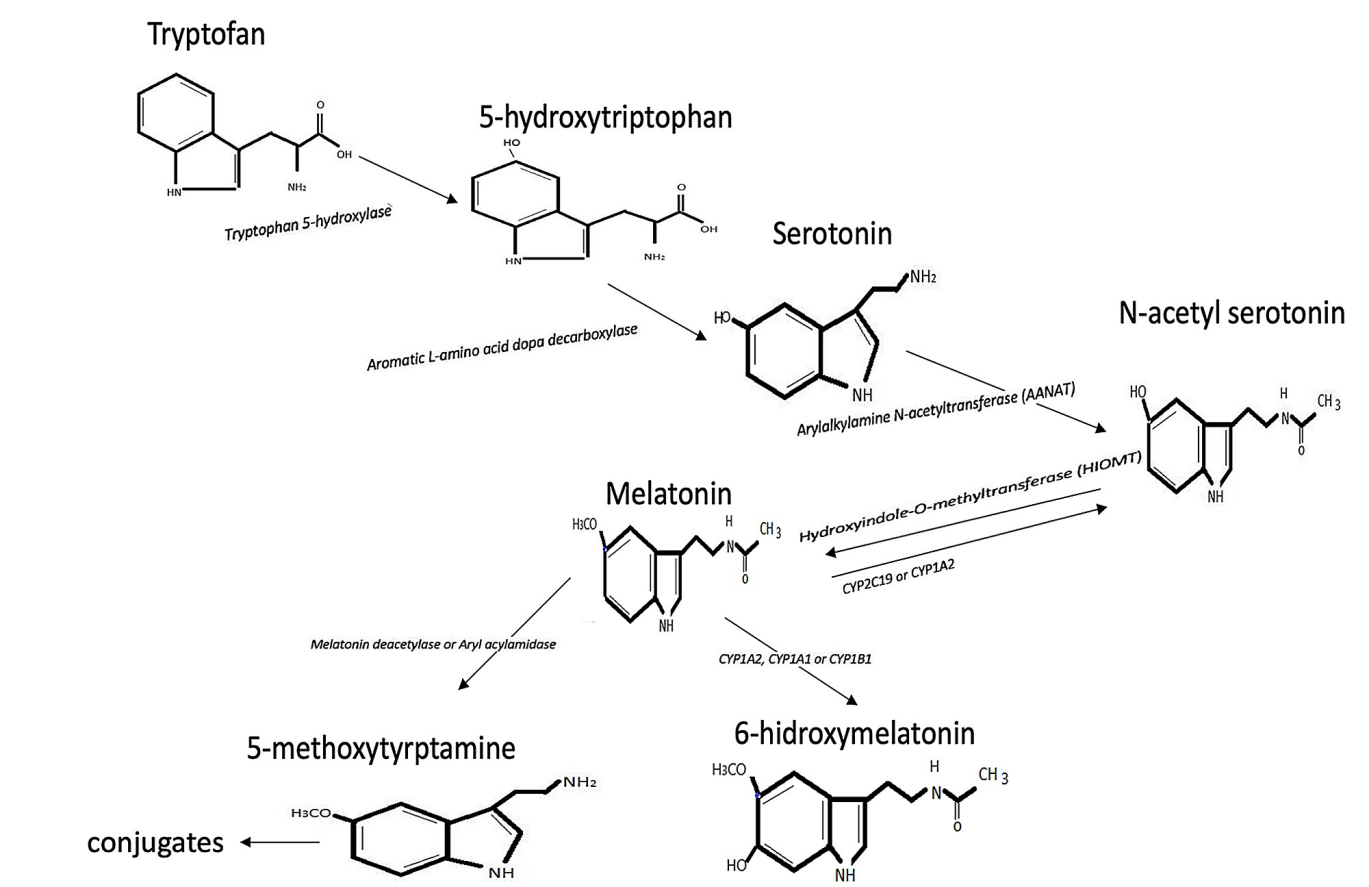
Melatonin metabolism.

Melatonin (N-acetyl-5-methoxytryptamine) is a neuroendocrine hormone, which was first isolated from the bovine pineal gland in 1958 by Lerner et al. [1]. The pineal gland was first determined by Herophilus in 325-280 BC. Pineal gland originates from pros-encephalon. It is innervated via superior cervical sympathetic ganglions, sphenopalatine and otic parasympathetic ganglions. Photic information is detected by photoreceptors in the retina and transferred to the suprachiasmatic nucleus (SCN) in the hypothalamus, paraventricular nucleus, the intermediolateral nucleus of the spine, and via sympathetic preganglionic adrenergic neurons to superior cervical ganglion respectively. Melatonin is synthesized from tryptophan via several enzymatic reactions [2]. These reactions occur in pinealocytes. The light/dark cycle is the main regulator of melatonin secretion. Norepinephrine stimulates melatonin synthesis and secretion via beta1-adrenoreceptors, stimulation of alfa1-adrenoreceptors potentiates the reaction. The rhythmic production of melatonin is controlled by the diurnal rhythm of activity of AA-NAT the rate-limiting enzyme. According to recent articles, a summary of melatonin metabolism is demonstrated in Figure 1 [3,4]. Melatonin is mainly secreted from the pineal gland; the other melatonin sources are retina, tissues of the gastrointestinal tract, skin, platelets, and bone marrow.

Melatonin’s pharmacokinetics

After intravenous or oral administration, melatonin is quickly metabolized, mainly in the liver and secondarily in kidneys. Melatonin is both lipid and water-soluble hormone which acts mainly through three types of high-affinity G-protein coupled receptors ML1 (Mel 1a) and ML2 (Mel 1b), and ML3. Mel 1a is a receptor that is mainly located on SCN and found to a lesser extent in the pituitary and cerebral vascular system. Mel 1b is the receptor type present in the retina. Ekmekcioglu et al. [5] showed melatonin receptors in coronary arteries. Besides CVS, melatonin receptors are multiple tissues. ML3 receptors are nuclear binding sites of melatonin [6], located in the cytosol, acts as an enzyme and responsible for detoxification of harmful agents. MT1 receptors are found mainly in CVS. Additionally, it can also be found in the immune system, placenta, retina, spleen, liver, breast, kidney, skin, testes, ovary, pancreas, adrenal cortex, retina and in the brain [4]. MT2 is found in the immune system, mammary glands, retina pituitary gland, adipose tissue, SCN, blood vessels, testes, gastrointestinal tract, kidney and skin.

The relationship between melatonin and blood pressure

Circadian variation of blood pressure and catecholamine levels were shown in several studies. Serum melatonin levels were found low in patients with hypertension [7]. The administration of exogenous melatonin decreases blood pressure both in hypertensive and normotensive patients [8,9]. Yıldız and Akdemir investigated the endogenous role of melatonin on arterial distensibility and blood pressure, for arterial distensibility assessed by aortic pulse wave velocity [10]. They showed a negative correlation between the velocity of the aortic pulse wave and di-urinal levels of melatonin was found [10]. In another study, melatonin was administrated orally, and its’ effect was compared to placebo in healthy male subjects [11]. Along with the reduction in catecholamine levels, a significant reduction of blood pressure was shown in the melatonin group [11]. Besides these findings, the pulsatility index in the internal carotid artery was decreased as well. In another randomized, double-blinded, placebo-controlled crossover trial, antihypertensive treatment native 16 male subjects were investigated for acute and repeated doses of melatonin [8]. Nocturnal systolic and diastolic blood pressure was reduced by 6 and 4 mmHg respectively in repeated melatonin doses. There was no change in heart rate [8]. In meta-analyses of randomized clinical trials on the effect of melatonin on blood pressure, it was shown that melatonin administration decreases both nocturnal systolic and diastolic pressure [9]. These effects might be contributed to the direct effect of melatonin via its’ receptor on the arterial wall or through modulation of autonomic activity [8,11]. Some investigators attribute the vasodilator effect of melatonin to its role in preventing the methylation of endothelial nitric oxide synthase [12]. Lusardi et al. [13] showed the chronic usage of melatonin in hypertensive patients well controlled by nifedipine therapy induces a blood pressure increase and a heart rate acceleration. They attributed this result to the competition between melatonin and nifedipine for the same calcium channels. Considering the melatonin receptors are seen on SCN, melatonin also provides feedback to SCN. There are conflicting data on the effect of melatonin on vascular tonus; these effects were assessed by Cook et al. in different vascular beds [14]. As a result, they found out that exogenous melatonin did not alter the mean arterial blood pressure comparing to the placebo group. Melatonin usage decreased renal blood flow and increased forearm blood flow. Melatonin usage did not alter cerebral blood flow. In the same study alpha adrenergic agonist administration reversed back the reduction in renal blood flow. This finding is explained by the favourable effect of melatonin on the sympathetic system. The mechanisms of the effect of melatonin on vasculature are explained in two pathways; receptor-mediated and via intercellular pathways [15,16]. In several studies melatonin binding to MT1 receptors on smooth muscle cells in vasculature results in vasoconstriction via inducing norepinephrine signalling [16]. Doolen et al. demonstrated a vasodilator effect on rat caudal arteries using a selective MT2 agonist 4-phenyl-2-acetamidotetraline [17]. There, seems to be a receptor-independent mechanism. Based upon the observations of Satake et al. and Vishwanathan et al., melatonin can also cause vasodilation without binding to its specific receptor [15,16]. Despite 125I-labeled melatonin binding was not demonstrated in rat aorta vasodilation was seen after melatonin injection. According to our current knowledge, MT1 receptor activation causes vasoconstriction; in contrast, MT2 receptor activation causes vasodilation [17]. The different responses of vascular bed to melatonin ingestion could be attributed to the distribution of different melatonin receptors.

The relationship between melatonin and lipid profile

There are several studies supporting the favourable effects of melatonin on lipid profile [18]. Behind the production and metabolism of lipoproteins, intestinal system and liver partake the most important role. To begin with fat metabolism, after digestion in the intestine, chylomicrons are transported from intestine to the liver. After several biochemical transformations, free fatty acids are converted to triglycerides (TG) and phospholipids in the liver. They are transported to blood by lipoproteins. LDL is the form of lipoprotein that carries the cholesterol to the cells also has a tendency to be oxidized by free oxygen radicals and cause damage to the cells and promote inflammation [19]. Several investigators focused on the damage caused by high reactive oxygen species (ROS) leading to atherosclerotic progression. In an animal study, investigators have demonstrated that melatonin supplementation with atherogenic diet increases atherosclerotic lesions in the proximal aorta in hypercholesterolemic mice via increasing the sensitivity of atherogenic lipoproteins to γ-radiolysis and Cu2+ oxidation in contrast to the majority of the other studies [19]. In another study, two weeks of melatonin treatment reduced free fatty acid levels significantly at cigarette smokers [20]. Another study performed on patients with non-alcoholic fatty liver disease, melatonin was administered for 14 months and demonstrated that LDL and triglyceride levels decreased in the melatonin group compared with the control group [21]. Melatonin administration decreased LDL-cholesterol levels besides blood pressure [18]. In a recent meta-analysis of eight randomized controlled trials, there was a significant association between melatonin supplementation and a reduction in total cholesterol levels, triglyceride levels [22]. But there was no significant effect on LDL and HDL levels. Melatonin having both lipophilic and hydrophilic properties is able to enter all types of cells and detoxify free oxygen radicals thereby inhibit LDL oxidation and damage caused by oxidizing LDL accumulation. Melatonin is found to protect the macromolecules from oxidation damage by its direct effect via inducing the antioxidant enzymes and its free radical scavenging effect. Favourable effects of melatonin on lipid profile could be attributed to the anti-inflammatory and anti-oxidative effects. Moreover, melatonin decreases lipid levels by enhancing the turnover of endogenous cholesterol into bile acids besides, inhibit cholesterol synthesis and accumulation. Still there need to be performed more randomized controlled trials to elucidate the underlying mechanisms of melatonin on lipid profile.

The relationship between melatonin, metabolic syndrome and diabetes mellitus

To our current knowledge, circadian rhythm interacts with endocrine metabolism. Sleeping disorders are associated with the development of type 2 DM and obesity [23]. Favourable effects of melatonin on the regulation of glucose metabolism are supported by several studies [24-32]. There are studies attributing to the positive effect of melatonin on end-organ damage in diabetic patients [24,25]. Ding M, et al. [24] demonstrated that melatonin diminishes the development of diabetes-induced cardiac dysfunction via avoiding mitochondrial fission through SIRT1- PGC 1α pathway. Emphasizing the regulatory role of melatonin on autophagy process melatonin may have favourable effects on diabetic retinopathy [25]. An in-vitro study on humans demonstrated that prolonged melatonin exposure of pancreatic islet cells turns out to improve glucose sensitivity [26]. Melatonin also reverses insulin resistance. In a study single nucleotide polymorphisms of melatonin receptors, was shown to be associated with impaired glucose tolerance and diabetes development [27]. The relationship between melatonin and insulin segregation, beyond these MT1 and MT2 receptors were detected in the pancreatic tissue. Some studies showed the suppressive effect of the pineal gland on pancreatic beta cell activity. Melatonin was associated with reduced glucose tolerance [28]. In an experimental study, melatonin administration to diabetic rats was not associated with hypoglycaemia or decreased levels of glycated haemoglobin but, decreased plasma bilirubin levels which attribute to the hepatoprotective properties of melatonin. Melatonin’s positive effects on oxidative stress are seen in diabetes mellitus. In a recent study on melatonin non-proficient 4 patients, Halpern B, et al. [29] demonstrated that after 3 months of melatonin supplementation fasting insulin levels and HOMA index decreased slightly in all patients. The predominant effect of melatonin on pancreas islets is a decline in insulin secretion [30]. In brief, melatonin inhibits insulin release from beta cells which is a protective mechanism for functional exhaustion of beta cells in type 2 diabetes, further-more, melatonin inhibits apoptosis and regenerates beta cells in type 1 diabetes [30]. In addition to these data showed, impaired glucose homeostasis and increased incidence of T2D was associated with single nucleotide polymorphisms in the MTNR1B locus in different ethnic backgrounds [31]. Several studies have shown an increased risk for DM in those subjects with MTNR1B gene mutation [32].

The relationship between melatonin and heart failure

Heart failure is a growing problem in the aging population all around the world. MT1 and MT 2 receptors were shown in cardiomyocytes. Although the exact role of melatonin in human ventricle function is unclear, two kinds of mechanisms are assumed to be involved in the action of melatonin in heart failure. These are receptor-dependent and receptor-independent mechanisms. Majority of the studies assessing the relationship between melatonin and heart failure emphasize on the protective effect of melatonin via its antioxidant properties rather than its direct effects through M1,2 receptor. Melatonin improves coronary flow and cardiac function through MT 1,2 receptors, beta adrenoceptors, and modulation of nitric oxide synthase (NOS). Another mechanism that leads to cell death thus heart failure is an ischemia-reperfusion injury which is a consequence of CAD [33,34]. Reperfusion injury occurs as a result of free oxygen radicals. Girotti et al. showed decreased melatonin levels among those patients admitted to the hospital with congestive heart failure and they concluded that lower melatonin levels lead to an exacerbation of congestive heart failure [35]. Tengattini et al. suggested that melatonin and nifedipine effect through the same calcium channels which are activated by KCL [36]. In one study it is claimed that melatonin can protect ischemia-reperfusion injury via activating silent information regulator 1 (SIRT 1) [37]. The protective effect of melatonin was attributed to its’ effect on increasing the antioxidant enzymes like Cu/Zn superoxide dismutase and stimulating phosphorylated protein kinase B (p-Akt) and inhibiting the activation of caspase cascade, therefore, inhibit apoptosis of mesenchymal cells [38]. The cytoprotective effect of melatonin depends on the time of administration of melatonin [34]. When given during the early stages of myocardial infarction, melatonin could prevent the progression of heart failure [34]. Injury due to ischemia and reperfusion is a result of free O2 radicals and their by-products, melatonin behaves as antioxidant and protects the myocardial tissue [35]. Melatonin is an electron-rich molecule. It may interact with free radicals to form metabolites that are also effective as free radical scavengers. Melatonin also activates several antioxidative enzymes including glutathione peroxidase (GSH), modulates gene expression for several protective enzymes and reduces lipid peroxidation. Additionally, the antioxidant effects of melatonin are probably based on its stimulatory effect on the expression of superoxide dismutase, GSH peroxidase, GSH reductase and glucose-6-phosphate dehydrogenase and its inhibitory effect on NOS expression. nitric oxide produces peroxynitrite and hydroxyl radicals which, in turn, induce peroxidation of membrane lipids and oxidation of other molecules [38]. Superoxide generation contributes to remodelling of ventricles in heart failure and as antioxidant melatonin seems to prevent myocardial remodelling. Melatonin is both lipophylic and hydrophilic. It diffuses easily into cellular compartments and provides on-site protection against free radical-mediated damage to biomolecules. Yeung et al. showed that melatonin administration to rats with chronic heart failure due to chronic intermittent hypoxia daily during four weeks, modulates calcium homeostasis while ischemia-reperfusion reduces inflammatory cytokines and fibrotic markers [39]. At the cellular level in non-ischemic heart failure melatonin reduces matrix deposition and fibrosis [40]. In a study on rats with isoproterenol-induced heart failure, Simko et al. investigated whether melatonin inhibits remodelling of the left ventricle and found that melatonin decreases the insoluble and total collagen and improved survival by modulating remodelling [41]. Melatonin may be a promising agent to prevent myocardial loss in acute myocardial infarction and thereby prevent heart failure development. 

The relationship between melatonin and cerebrovascular disease

There are several studies highlighting the protective effect of melatonin in the central nerve system. Stroke is caused by occlusion or haemorrhage of one of the cerebral arteries perfusing the brain tissues. Reperfusion of the ischemic phase conduces to the production of free oxygen radical production, which is the responsible mechanism of brain cell damage. There are several mechanisms that contribute to the protective effect of melatonin on neuronal cells. Melatonin reduces the oxidative stress via scavenging free oxygen radicals and inducing the gene transcription of anti-oxidative enzymes. Melatonin is shown to reduce the mitochondrial permeability which is seen in the pathophysiology of cell damage in stroke and inhibits the release of death factors into the cytosol and thus cell death [42,43]. Melatonin has anti-apoptotic and anti-inflammatory effects [44]. According to our present knowledge, melatonin has a favourable effect on cerebrovascular system primarily via its’ antioxidant character. In one study, melatonin effect was investigated among rats with acute stroke, they found that rats that have received rat-derivate pineal gland have decreased in the infarcted area in comparison to those that did not receive any transplants and developed better motor skills [45]. In another study performed on mice that administrating (5 mg/kg) melatonin at the beginning of reperfusion decreases ischemic injury of grey and white matter [46]. Melatonin has a favourable effect on cerebral edema formation among those animals treated for stroke [47].

The relationship between melatonin and CAD 

**Figure 2:**
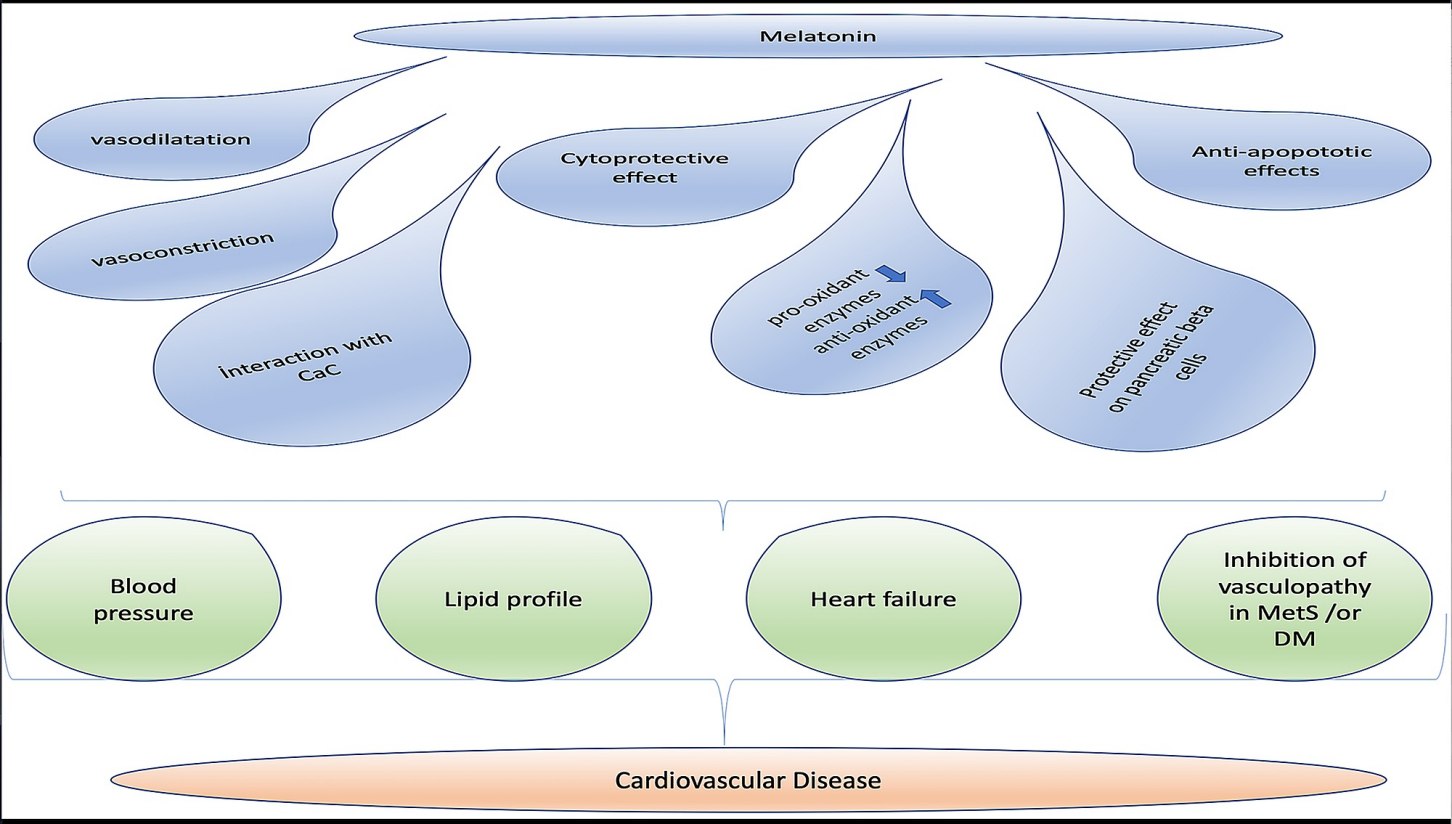
Effects of melatonin. MetS: metabolic syndrome; DM: diabetes mellitus.

CAD is the major cause of death and morbidity all around the world. Atherosclerotic plaque formation and progression could be summarised in following steps; lipid deposition, exposure to oxidative stress, inflammation, endothelial dysfunction, and vascular smooth muscle cell differentiation and atherosclerotic plaque formation. Investigators claim that damage caused by high ROS leads to inflammation, endothelial dysfunction and finally atherosclerotic progression. Melatonin contributes to atherosclerosis via affecting the factors which contribute to the pathogenesis of plaque formation and rupture such as high blood pressure, dyslipidaemia, sympathetic predominance, inflammatory process [48]. Melatonin having both lipophilic and hydrophilic properties is able to enter all types of cells and detoxify free oxygen radicals [38]. By its direct effect on inducing the antioxidant enzymes and its free radical scavenging effect; it protects the macromolecules from oxidative stress. In another study performed on those patients with normal coronaries and those with CAD, although there was a large inter-individual variation in the pattern of the secretion of melatonin, it was found that nocturnal secretion of melatonin was decreased in patients with CAD when compared to healthy subjects [49]. Additionally, Altun A et al. demonstrated decreased nocturnal melatonin levels in patients with cardiac syndrome X [50]. The pathophysiology of cardiac syndrome X includes impaired baroreceptor sensitivity and sympathetic predominance, which causes reduced responsiveness to an adrenergic stimulus which may also influence the pineal gland and decrease melatonin synthesis. Yıldız et al. demonstrated a decreased velocity of the aortic pulse wave, blood pressure, and heart rate. The velocity of the pulse wave is one of the well-known indicators to detect patients at high cardiovascular risk. Important factors contributing to its’ increase in the human population is age, increased arterial stiffness due to medial calcification, increases in collagenous material, and loss of arterial elasticity. The velocity of the wave is higher at high blood pressure and reflects heart rate and sympathetic stimulation [10]. In a recent study, melatonin was tested to detect whether it improves ischemia in patients with ST-elevation MI [33]. In this study, the infarct size expressed by the peak of troponin I in 24 hours was significantly smaller than the placebo group. Like-wise calculation of left ventricular mass by CMR showed better outcomes in the melatonin group compared with the placebo group. Those patients who had revascularized and received melatonin in the early phase of MI, showed decreased infarct size. However, those who were revascularized and received melatonin in late phase of the infarction, developed larger infarct area. According to this study, it can be concluded that timing of administering melatonin is an important issue for avoiding myocardial injury [33]. There are several studies that claim the cytoprotective effect of melatonin administered during the early stages of myocardial infarction thus prevent the progression of heart failure [33,34]. Considering the role of inflammation, in CAD in addition to hyperlipidaemia, hypertension, advanced age and gender; via its’ antioxidant features in addition to its favourable effect on smooth muscle cells and autonomous nerve system, melatonin has a protective effect on CVS. Despite the majority of studies about the favourable effects of melatonin on atherosclerosis, there are studies indicating the unfavourable effect on the atherosclerotic plaque [19]. The effects of melatonin in multiple systems are briefly demonstrated in Figure 2. 

## Conclusions

Melatonin effect on the cardiovascular system seems to be fundamentally based on its favourable effects on lipid and glucose metabolism besides blood pressure and sympathetic system. Although there are conflicting data on the effect of melatonin on vascular tonus, the majority of the studies have shown the favourable effects of melatonin on blood pressure. According to our current knowledge, MT1 receptor activation causes vasoconstriction; in contrast, MT2 receptor activation causes vasodilation. Also, the direct effects of melatonin are observed on potassium channels. The different responses of vascular bed to melatonin ingestion could be attributed to the distribution of different melatonin receptors. Still, the majority of studies have claimed the decrease in blood pressure among those subjects given melatonin. Melatonin, having both lipophilic and hydrophilic properties is able to enter all types of cells and detoxify free oxygen radicals thereby inhibit LDL oxidation and damage caused by oxidizing LDL accumulation. Moreover, according to some investigators hypocholesterolaemia effect of melatonin is attributed to its’ role in enhancing the clearance of cholesterol and reducing its’ synthesis. Improved glucose tolerance in humans, has been identified with prolonged melatonin exposure. Several studies demonstrated the relationship between melatonin and insulin segregation, beyond these MT1 and MT2 receptors were detected in the pancreatic tissue. Melatonin improves glucose sensitivity by regulating the growth of pancreatic islet cells. Melatonin is shown to reduce the mitochondrial permeability which is seen in the pathophysiology of cell damage in stroke and inhibits the release of death factors into the cytosol and thus cell death. Therefore, it may be speculated that due to its anti-oxidative and cytoprotective features melatonin given, may limit the neuronal cell loss in those patients suffering an acute ischemic stroke. The antioxidant effect is the primary mechanism of melatonin in heart failure. Especially in MI, the mechanism that leads to cell death thus heart failure is an ischemia-reperfusion injury. Reperfusion injury occurs as a result of free oxygen radicals. Depending on the timing and dosage administrated, melatonin can prevent the progression of heart failure. Melatonin shows its antioxidant effect rather than its direct effects through M1, 2 receptors. Besides increasing the antioxidant enzymes, it inhibits the apoptosis of mesenchymal cells. The coronary atherosclerotic process involves different pathological mechanisms; inflammation is one of the major triggers for the development of atherosclerotic plaque: lipid deposition, exposure to oxidative stress, inflammation, endothelial dysfunction, and vascular smooth muscle cell differentiation and atherosclerotic plaque formation. Factors such as an increased blood pressure and diabetes mellitus also cause endothelial dysfunction. According to the majority of the trials, melatonin as an agent which takes a positive effect on both circadian rhythm, insulin resistance, lipid profile may act as a favourable mediator for CAD management. Despite contradictions between the studies the majority of data claims that melatonin is a promising supplement with hardly any adverse effects. To assess its effect more clearly there need to be prospectively designed human trials with a large sample size.

.
